# Bring in the genes: genetic-ecophysiological modeling of the adaptive response of trees to environmental change. With application to the annual cycle

**DOI:** 10.3389/fpls.2014.00742

**Published:** 2015-01-13

**Authors:** Koen Kramer, Bert van der Werf, Mart-Jan Schelhaas

**Affiliations:** Alterra - Green World Research, Vegetation, Forest and Landscape Ecology, Wageningen University and Research CentreWageningen, Netherlands

**Keywords:** adaptation, adaptive capacity, ForGEM, genetic diversity, modeling, phenology

## Abstract

The observation of strong latitudinal clines in the date of bud burst of tree species indicate that populations of these species are genetically adapted to local environmental conditions. Existing phenological models rarely address this clinal variation, so that adaptive responses of tree populations to changes in environmental conditions are not taken into account, e.g., in models on species distributions that use phenological sub-models. This omission of simulating adaptive response in tree models may over- or underestimate the effects of climate change on tree species distributions, as well as the impacts of climate change on tree growth and productivity. Here, we present an approach to model the adaptive response of traits to environmental change based on an integrated process-based eco-physiological and quantitative genetic model of adaptive traits. Thus, the parameter values of phenological traits are expressed in genetic terms (allele effects and—frequencies, number of loci) for individual trees. These individual trees thereby differ in their ability to acquire resources, grow and reproduce as described by the process-based model, leading to differential survival. Differential survival is thus the consequence of both differences in parameters values and their genetic composition. By simulating recombination and dispersal of pollen, the genetic composition of the offspring will differ from that of their parents. Over time, the distribution of both trait values and the frequency of the underlying alleles in the population change as a consequence of changes in environmental drivers leading to adaptation of trees to local environmental conditions. This approach is applied to an individual-tree growth model that includes a phenological model on the annual cycle of trees whose parameters are allowed to adapt. An example of the adaptive response of the onset of the growing season across Europe is presented.

## Introduction

Genetic diversity is the ultimate source based on which species adapt to climate change (Geburek and Turok, 2005). Evolution resulted in the adaptation of plant species to local climatological conditions and consequently they respond differently to climate change. Also within plant species, local adaptation has occurred over time. Transplantation trials of tree species throughout Europe have shown that provenances, transferred within the geographic range of the species, differ in degree and even in sign of their response to changes in precipitation and temperature (Mátyás, 1996; Rehfeldt et al., 2002; Alberto et al., 2013). This genetic diversity within a species, as a result of adaptation to local environmental conditions, is important at the limits of species distributions (Hampe and Petit, 2005). Genetic diversity is typically lowest at the expanding front of the species' distribution and highest at the retreating limit, thereby affecting the survival of the individual trees and thus the rates of expansion and retreat, respectively (Petit and Hampe, 2006). In the center of the species distribution, it is particularly the vulnerability to extreme events and the capacity to recover from these events, where genetic diversity within a species plays an important role (Bengtsson et al., 2000; Parmesan et al., 2000).

Management can have a major impact on the genetic diversity of perennial plant species (Valladares, 2008). Selection aiming at maximization of productivity of forest- and fruit trees and nut-bearing trees reduces genetic diversity. Also management measures to mitigate climate change impacts by means of assisted migration outside the existing species range, may decrease the capacity of the species to adapt to on-going climate changes because of a too low initial genetic diversity (McLachlan et al., 2007; Leech et al., 2011). Current climate change assessment modeling ignores local adaptation of long living perennial plant species, such as trees. In this paper we argue that adaptive response in a genetic sense is an important issue that needs to be included in climate change impact assessment studies. We indicate how adaptive capacity and adaptation can be included in process-based models to attain more accurate local predictions over large spatial range.

The structure of this paper is as follows. Firstly, general quantitative genetic issues of adaptive capacity and adaptive responses are presented, followed by a presentation how these quantitative genetic issues are brought in a classical ecophysiological approach of individual tree modeling. Technical aspects and derivations of the key-genetic equations used in the coupled genetic—ecophysiological model are placed in the Supplementary Materials. Secondly, a general approach of characterizing a forest stand is presented, followed by a description of the stand characterization as used in the model analyses presented in this study. The subsequent sections presents, discusses and draws conclusions on the simulated results, respectively. Parts of this model description are also presented in Kramer et al. (2013) and Kramer et al. (2008) but repeated here to have a full account of the genetic model. See Kramer et al. (2008) for references for the ecophysiological part of the model.

## Modeling adaptive capacity and adaptation

### Quantitative genetics

Adaptation is the dynamic evolutionary process that leads to a trait becoming adapted to local environmental conditions by means of natural selection, i.e., differential survival as a consequence of differences in values of the trait under selection. Adaptive capacity in its genetic sense, is the potential of a population to respond to an environmental change by having its genetic composition modified and, as a consequence, also the phenotypic expression of functional traits. The population thereby becomes better adapted to the new environmental conditions. The adaptive response thus refers to the actual change in genetic composition and thereby the value of the functional trait.

Quantitative genetics is the part of genetics that studies polygenic traits, i.e., traits that are under the influence of many loci (i.e., the location of the genetic information of a trait on the DNA string), each locus with two to many alleles (i.e., variation in the genetic information for that locus in the population). As there are many loci and potentially many alleles, the contribution of a single locus and allele on the phenotypic expression of the trait is only small. The contribution of the alleles and loci to the phenotypic values of a trait can be partitioned into additive, dominant (allele × allele interactions), epistasis (locus × locus interactions) and a remaining non-genetic component (Falconer and Mackay, 1996). Quantitative genetic studies are often restricted to additive effects because this is the component being inherited, and the determination of dominances and epistasis requires extensive experimental designs. As the additive allelic effects are considered constant, a particular combination of alleles over the loci determine the genotypic value of the traits, which, enlarged with the environmental component, defines the phenotypic value of a trait for an individual organism. Differential survival as a consequence of climate change, results in changes in the frequency of the alleles and thereby a change of the distribution of phenotypic values of a population. Thus, the population adapts to local environmental conditions. As a consequence of adaptation, some alleles will be lost from the population, either because these allelic effects are unfavorable under the new conditions or because of genetic drift. This loss in genetic diversity results in a reduced adaptive capacity to future environmental changes. Genetic processes to increase genetic diversity of adaptive traits are immigration of genetic material by gene flow from other populations, and mutation. In case of perennial plants, gene flow means input of pollen and seeds, or planting of new genetic material. Considering mutation, the low natural rate of mutation makes that this is in a time frame of a few generations relevant only for very large randomly mating populations.

### Bridging eco-physiology and quantitative genetics in plant models

**Figure 1:**
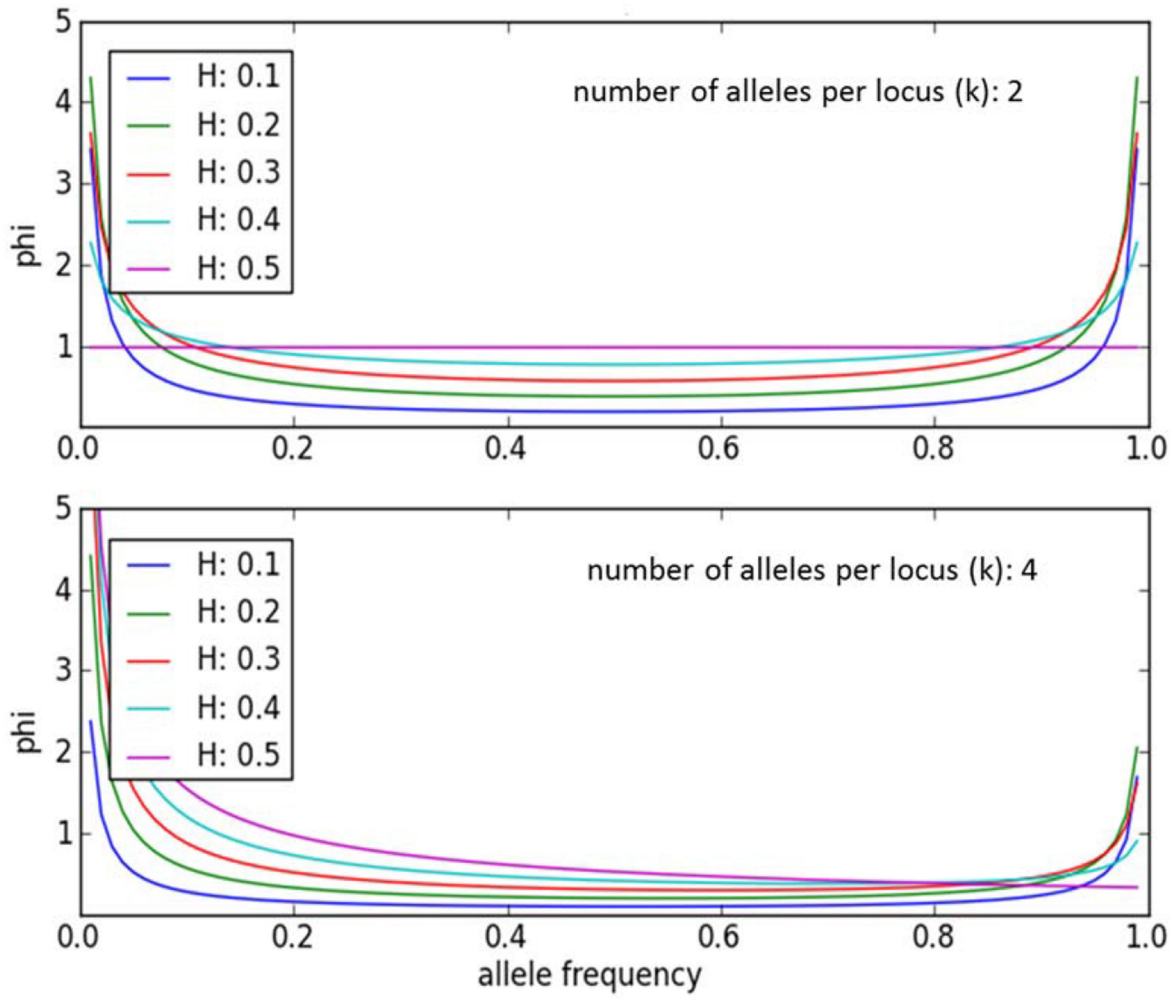
**Equilibrium allele frequency distribution, *phi*, for different values of heterozygosity (*H*) and number of alleles per locus *k* (Nei, 1987)**. Most alleles have either a very low or a very high frequency, whereas few alleles have a frequency around 0.5. Except when *k* = 2 and *H* = 0.5. Under those conditions *phi* = 1.

**Figure 2:**
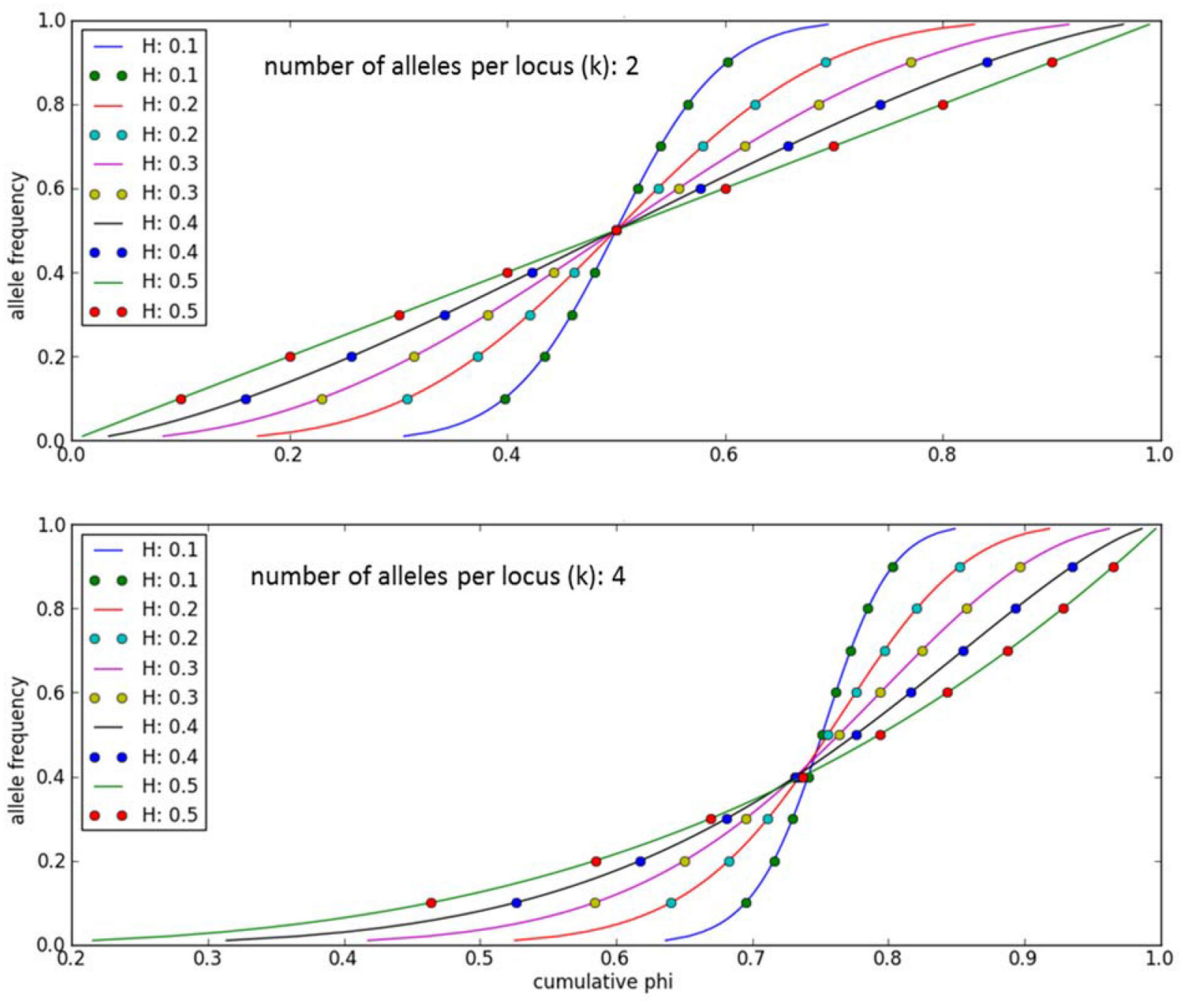
**Allele frequencies to initialize the ForGEM model for different values of heterozygosity (*H*) and number of alleles per locus (*k*)**. The dots indicate the allelic effects for a 10-locus trait evenly spaced over cumulative *phi(x)*. The same cumulative distribution of *phi* can be used if a trait is determined by another number of di-allelic loci.

**Figure 3:**
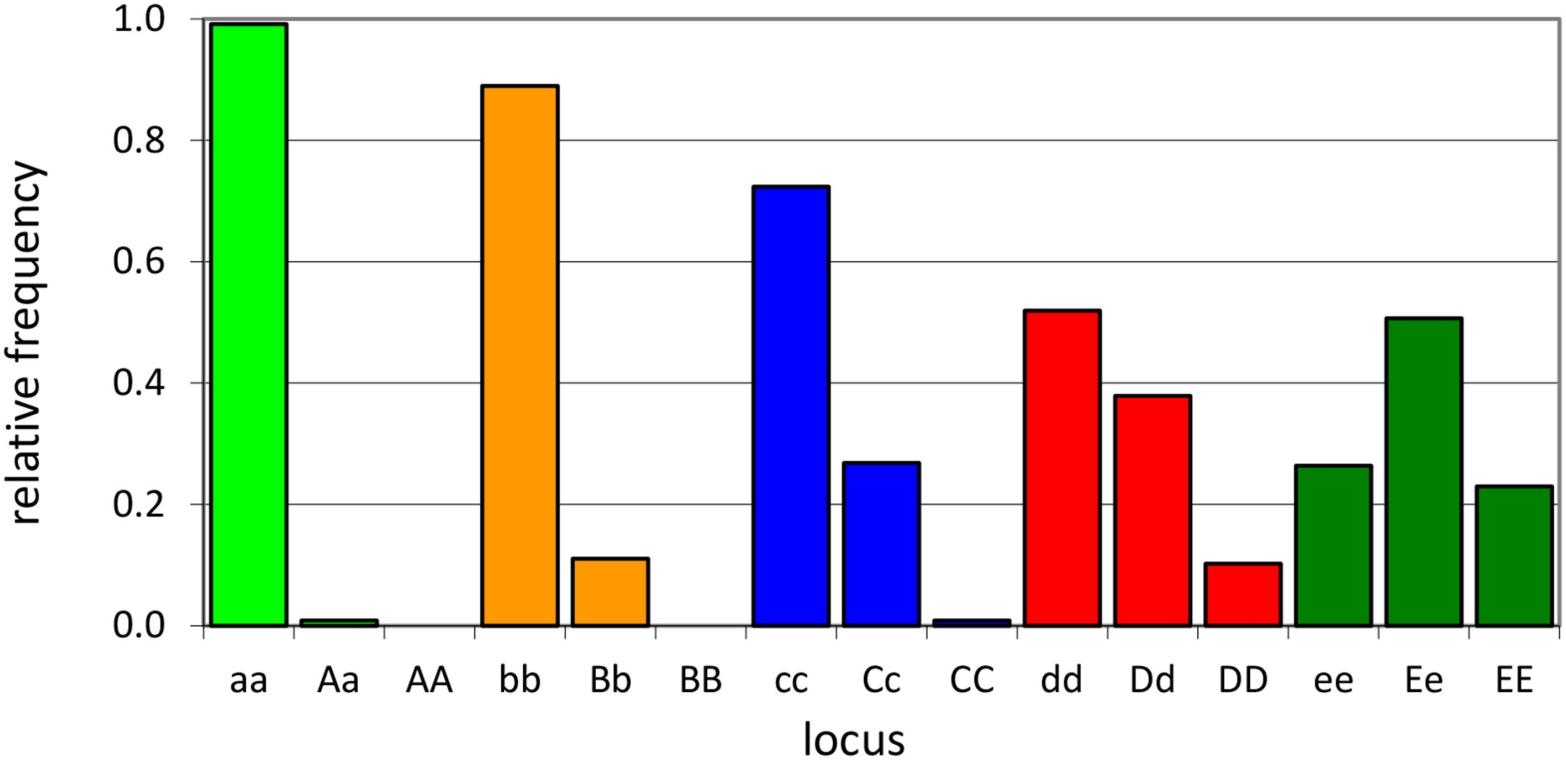
**Example of initial genotype relative frequency distribution for a 5-locus, 2-allele genetic system (see A1 of the Supplementary Material for the allele frequencies used)**.

An individual-plant model in which process-based modeling is connected to a quantitative genetic representation of eco-physiological parameters is the ForGEM model (Forest Genetics, Ecophysiology, and Management) (Kramer et al., 2008; Kramer and van der Werf, 2010). In principle each of the model parameters can be characterized by the genetic model and evolve due to environmental change. The genetic system can be initialized, i.e., setting initial allele *frequencies* and assigning allelic *effects*, either by taking a statistical approach or by using observed allele frequencies and allelic effects for Quantitative Trait Loci (QTLs), Candidate Genes (CGs) or actual genes, determined in experimental populations (Brendel et al., 2008). As the initial distribution of allele frequencies has a strong effect on the simulated rate of the adaptive response, we assume, based on theoretical considerations, that initially the allele frequency distribution follows a U-shaped beta distribution, *phi*. (Figure 1) (Gillespie, 2004). That allele frequency distribution is a function of the heterozygosity of the traits (*H*) and the number of alleles (*k*) (Nei, 1987). Inverting the cumulative distribution of φ leads to the initial allele frequencies (Figure 2, see Appendix B in Supplementary Material and Kramer et al. (2008) for details). Reasonable values for quantitative traits are: number of loci = 10, *H* = 0.25, and *k* = 2 (Kramer et al., 2008). Multiplication of all combinations of initial allele frequencies leads to initial genotypic frequencies (Figure 3, for brevity in this example a 5-locus, 2 allele system is presented).

**Figure 4:**
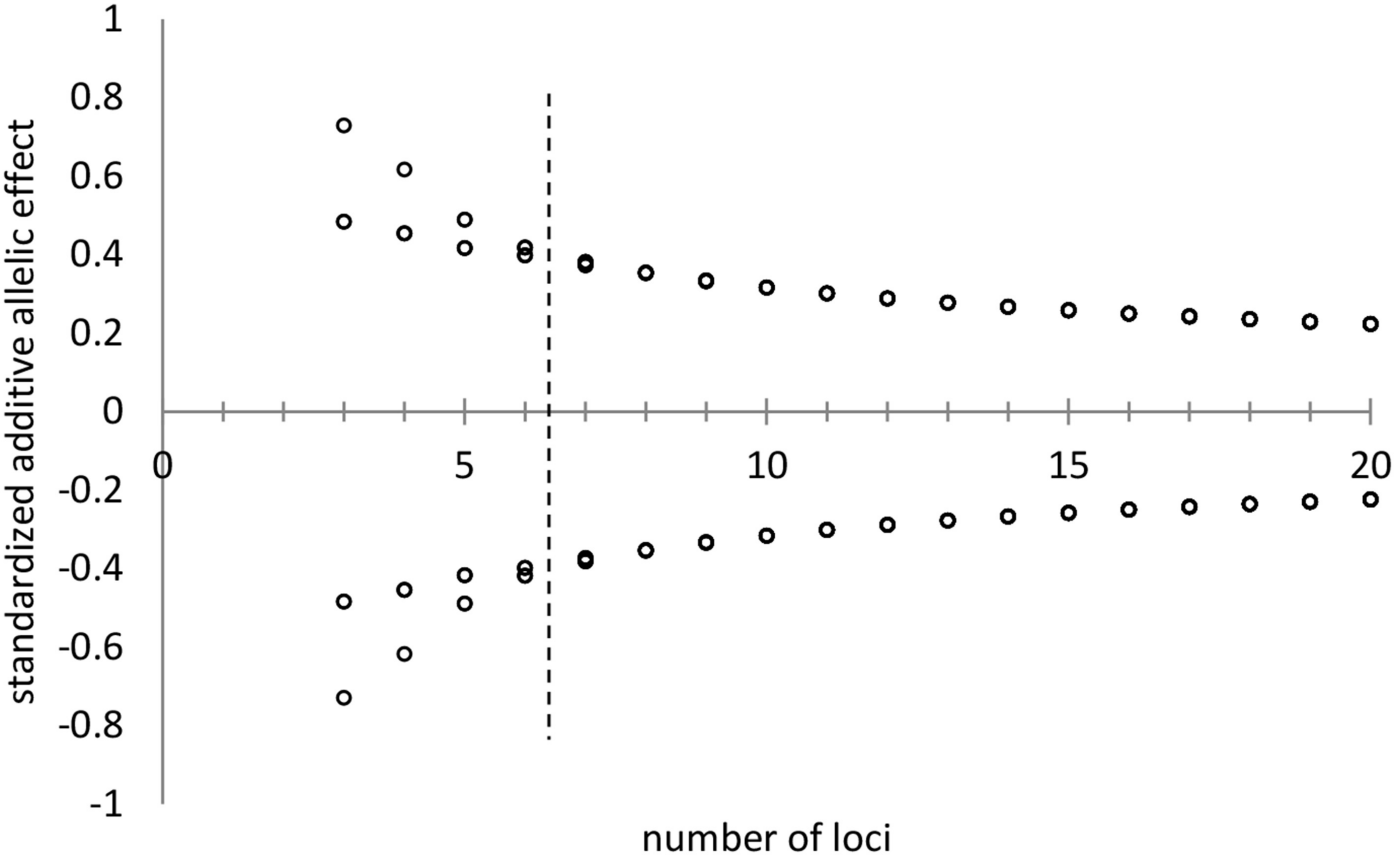
**Standardized additive allelic effects (i.e., mean is zero, variance is unity) assigned to di-allelic multi-locus traits, under the constraint of the distribution of allelic frequencies as indicated in Figures 1, 2 with *k* = 2 and *H* = 0.25**. With a low number of loci (number of loci < 7, left of the hashed line) two symmetric allelic effects are attained. At higher values for the number of loci per trait, all alleles have virtually the same effect on the genotype (right of the hashed line).

**Figure 5:**
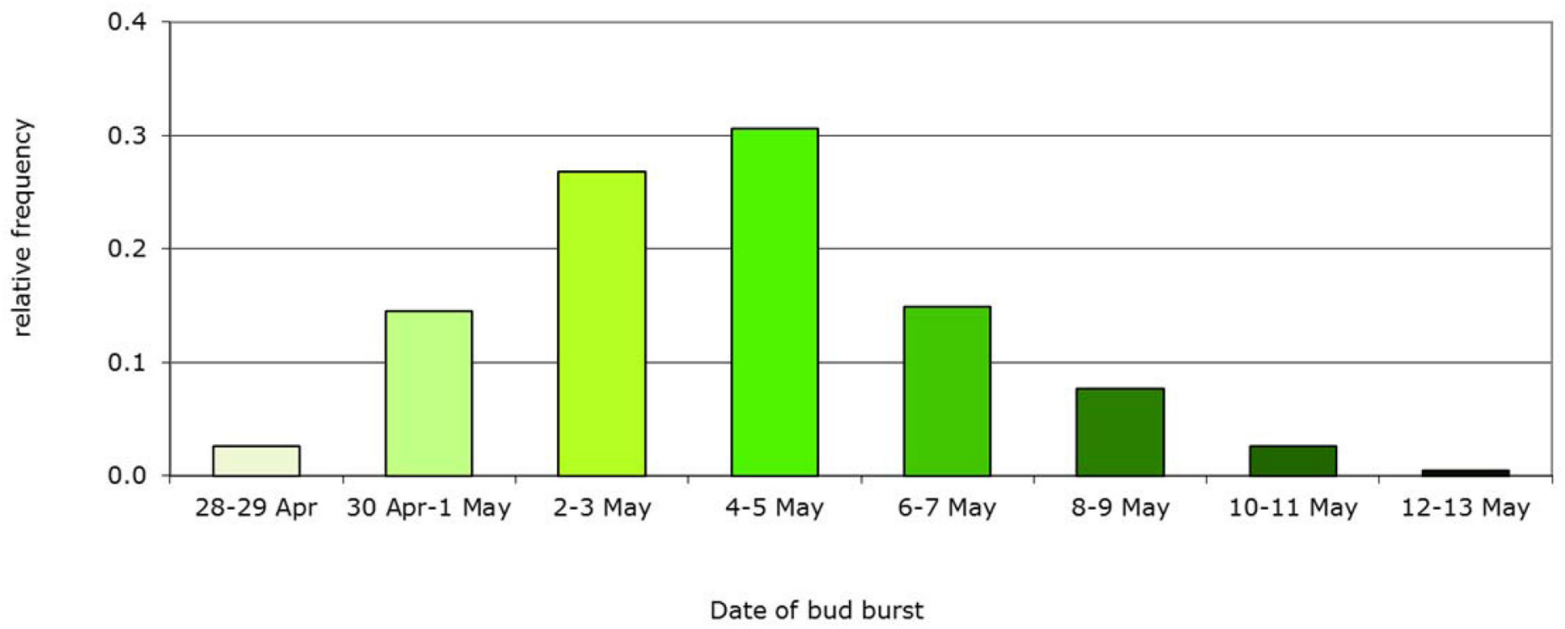
**Example of initial frequency distribution of a phenotypic trait: bud burst date based on the Dutch beech population**. Mean bud burst date May 5th, genetic variance *V_g_* = 5 d, population, *n* = 235 trees.

The allelic effect of a particular allele is the deviate from the population mean of the genotypes that possess that allele. The deviate is expressed in units standard deviation of the genetic variance. Allelic effects are determined in the ForGEM model by first assigning +1 and −1 values to the two alleles of di-allelic multi-locus traits and subsequently normalizing the allelic effects (mean of zero, variance of unity) under the constraint of the U-shaped distribution of allelic frequencies as indicated above. Figure 4 shows the decline in allelic effect with increasing number of loci for a di-allelic genetic system with symmetric allelic effects. Genotypic values for a trait (i.e., model parameter value) are attained by summing the normalized allelic effects of the genotype multiplied with the genetic standard deviation and adding the observed population mean of the trait (see the Supplementary Material for details). Phenotypic values are attained by enhancing the genotypic values with an environmental deviate based on the heritability of the trait. For example, the genotypic values of the trait range from 20*E* to -20*E* in the population in case of a 10 locus, di-allele system, with *E* representing the allelic effect. Figure 5 presents an example of this approach for the outcome of the distribution of bud burst dates. See Appendix A in Supplementary Material for details on initial allele frequencies and attaining genotypic and phenotypic values, with a numerical example, and (Kramer et al., 2008) for sensitivity analyses of the model.

### General aspects for characterization of forest stands

**Figure 6:**
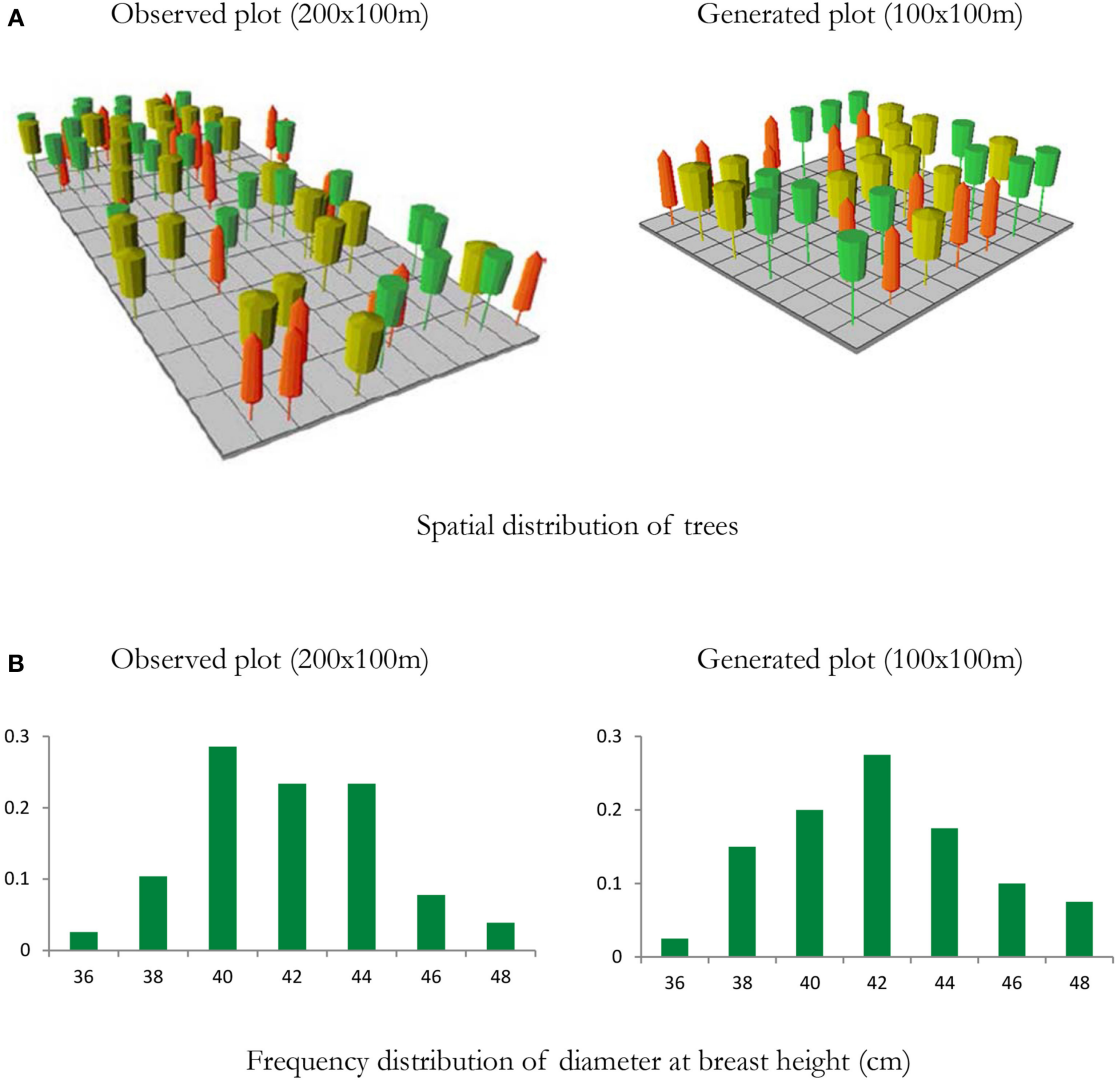
**Example of a mixed species forest stand to initialize the ForGEM model**. Spatial distribution of trees and diameter distribution of a 2 ha observed plot from a national forest inventory with individually measured trees, and a representation of a 1 ha generated plot based on stand statistics of the observed plot (density per species, mean and coefficient of variation of height and diameter at breast height). **(A)** Visualization of the stand structure of the observed and generated plots, **(B)** distribution of DBH of observed and generated plot. Note that spatial structure is not accounted for in the generated plot. Yellow trees—*Quercus robur*; Orange trees—*Fagus sylvatica*; Green trees—*Fraxinus excelsior*. Visualized with Stand Visualization System SVS, (McGaughey, 1997). In the model analyses presented below, a pure beech forest was generated with this approach.

The ForGEM model is an individual-tree model. If no individual-tree measurements of a stand are available, observed stand information from National Forest Inventories (NFI) can be used to initialize the stand. Typical NFI information are mean height and diameter of the different tree species occurring on a plot, possible including a measure of variability. The statistics of the NFI plot are then used to generate a forest stand with statistically the same characteristics (see Figure 6 for an example of a mixed species forest). For forest management, we follow the classification of Forest Management Approach (FMA, Table 1, Duncker et al., 2012). This approach can accommodate scenario assumptions on changes in forest management due to policy and market developments. Daily meteorological parameters are required. Which were obtained from the meteorological database provided by ISI-MIP, including climate change scenarios. Both the NFI and meteorological data and the distribution of FMA's over Europe are available so that the ForGEM model can be run throughout Europe with a resolution of 1 × 1 km.

**Table 1:** **Characterization of Forest Management Approaches (FMAs) (Duncker et al., 2012)**.

**FMA**	**Title**	**Management intensity**	**Objective**
1	Unmanaged forest/nature reserve	Passive	To allow natural processes and natural disturbance regimes to develop without management intervention
2	Close-to-nature forestry	Low	To manage a stand with the emulation of natural processes as a guiding principle; any management intervention in the forest has to enhance or conserve the ecological functions of the forest
3	Combined objective forestry	Medium	A mix of different objectives, additional objectives to timber production can be water and soil protection, mushroom production, habitat protection, avalanche prevention, game management and nature protection, fire prevention and/or recreation, and are adapted to the local situation
4	Intensive even-aged forestry	High	To produce timber
5	Short rotation forestry	Intensive	To produce the highest amount of merchantable timber or wood biomass

### Characterization of forest stand for model analyses presented

As example we analyzed the adaptive response of phenological parameters for sites located in different environments. The source population was a Dutch beech population for which the values of a phenological model developed and tested for beech were available (Kramer, 1994a,b). The critical state of chilling (S^*^_c_) and the critical state of forcing (S^*^_f_) were used as parameters whose distribution of values in the population are allowed to change in the population based on the genetic system described above. All the other model parameters were kept constant. The penalty of a too early bud burst is a loss of the foliage and flowers of adult tree if the temperature after the bud burst day is less than the level of frost hardiness of the tree (−2°C for deciduous broadleaves, Kramer et al., 2008), and death of all seedlings with emerged leaves. The gain of an earlier bud burst is an increase of productivity as described by the process-based model. Thus, there is a trade-off between: (i) a too-late bud burst, resulting in not capturing the available resources during the growing season and increased mortality relative to individual trees that do capture these resources; and (ii) a too early bud burst, resulting in a loss of offspring and increased cost for rebuilding the canopy. See Appendix C in Supplementary Material for a description of how mortality is determined by the model.

**Figure 7:**
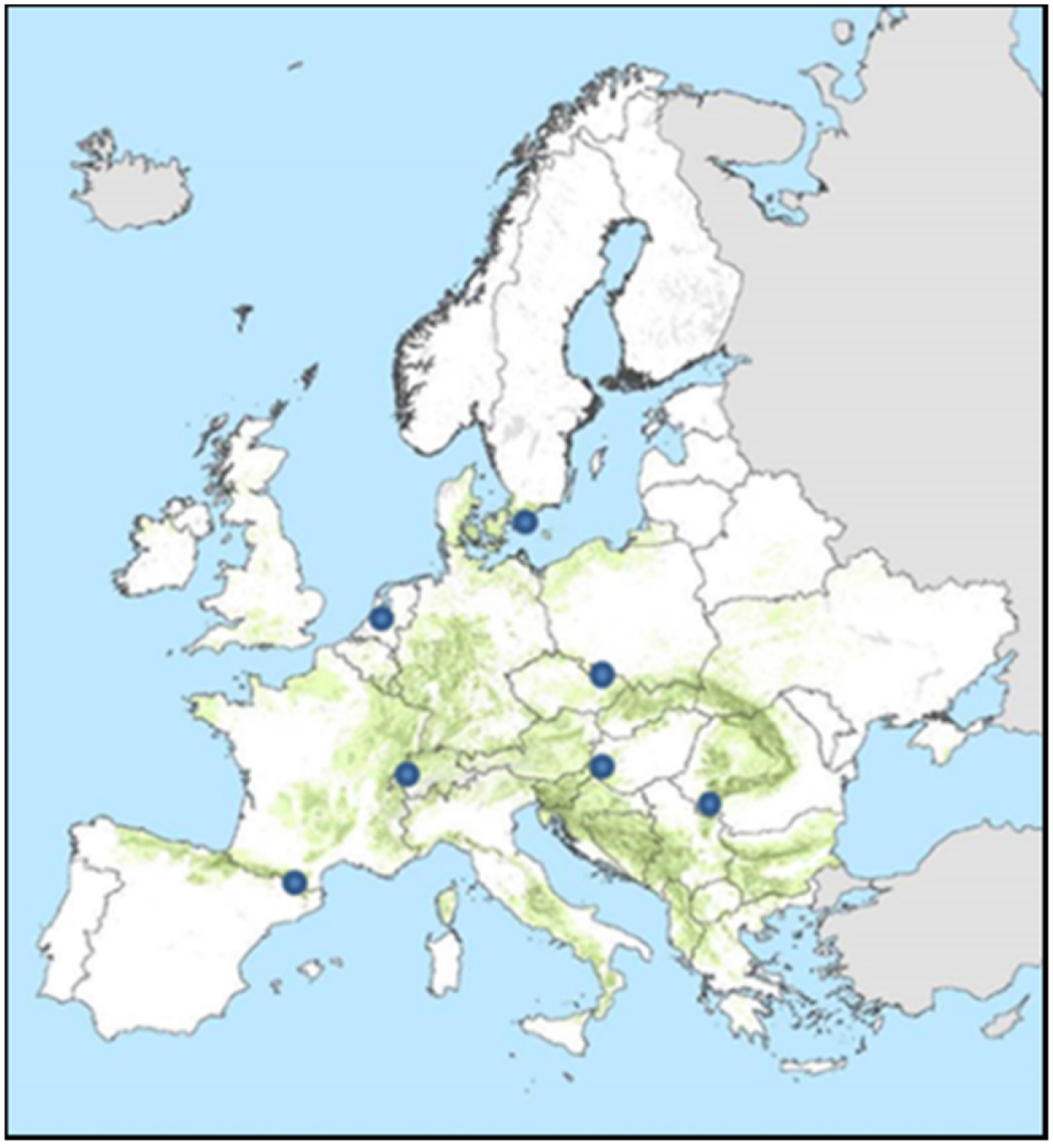
**Location of the sites for which the ForGEM model was run**. Green indicates the distribution of beech in Europe (Brus et al., 2012).

Pure beech stands at different locations in Europe (Figure 7, Table 2) were initialized with the same genetic composition (distribution of allelic effects over 10 di-allelic loci) as the Dutch population. The initial stand was generated based on a yield class of 12 m^−1^ ha^−1^ yr^−1^ (Jansen et al., 1996). The initial stem density was 581 trees ha^−1^, average height 18.6 m (coefficient of variation = 0.1), average dbh 18.5 cm (coefficient of variation = 0.1), age 40 years. To account for the stochasticity in the model, 5 replicates were simulated. New initial stands were generated for each replica based on the coefficient of variation for height and dbh. Locally representative weather data were randomly sampled +2°C scenario from ISI-MIP database (hadgem2-es_rcp2p6, ISI-MIP^1^) The different replicate runs thus differ both in weather and initial stand structure (i.e., height and dbh and position of the trees in the stand). An intensive even-aged forestry system was applied at all locations (FMA 4 from Table 1). This system consists of a seed-tree cut at a stand age of 60, where the tree density is decreased to 50 trees per hectare. Trees lower than 5 m are retained. The seed trees are harvested at stand age 70. The age of the stand is calculated as the average age of all trees that have reached at least 50% of the maximum height.

**Table 2:** **Characterization of the sites for which the ForGEM model was run**.

**Country**	**Latitude**	**Longitude**	**Radiation MJ m^−2^ yr^−1^**	**Precipitation mm yr^−1^**	**Temperature (avg. [min, max]) C**
Czech Republic	50.25	17.25	3295	1127	8.8 [−15.8, 34.7]
Hungary	46.75	16.75	4031	758	13.4 [−11.2, 38.8]
Netherlands	52.25	5.25	3333	876	11.3 [−7.1, 34.1]
Romania	45.25	23.25	3971	836	10.7 [−14.6, 34.4]
Spain	42.75	1.75	4630	989	10.2 [−9.5, 31.8]
Sweden	56.25	14.25	3368	720	8.9 [−10.9, 31.5]
Switzerland	46.75	6.75	3775	1304	10.7 [−10.3, 33.5]

*Min, max for temperature indicate the average over 22 years of the lowest minimum and maximum temperature, respectively. Meteorological data obtained from ISI-MIP project, using the scenario of the Hadley Global Circulation Model (Pope et al., 2000) that aims at a 2°C temperature increase*.

## Results

**Figure 8:**
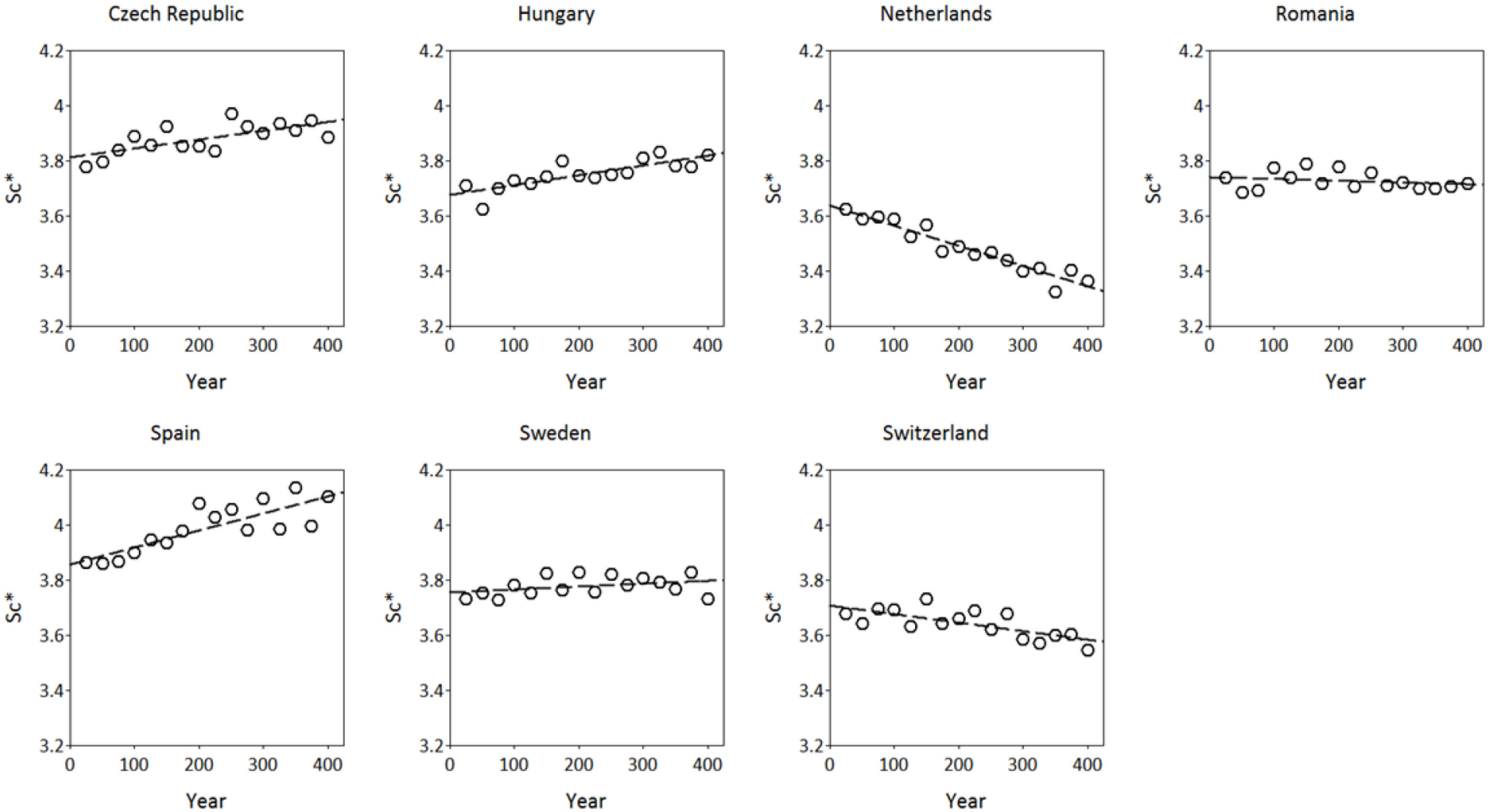
Adaptive response of critical state of chilling (S^*^_c_, in chilling units, see Kramer, 1994b) over time after translocation of the Dutch population to the country indicated. The results presented are the mean of 4 replicate runs to account for the genetic and environmental stochasticity (one stand died).

**Figure 9:**
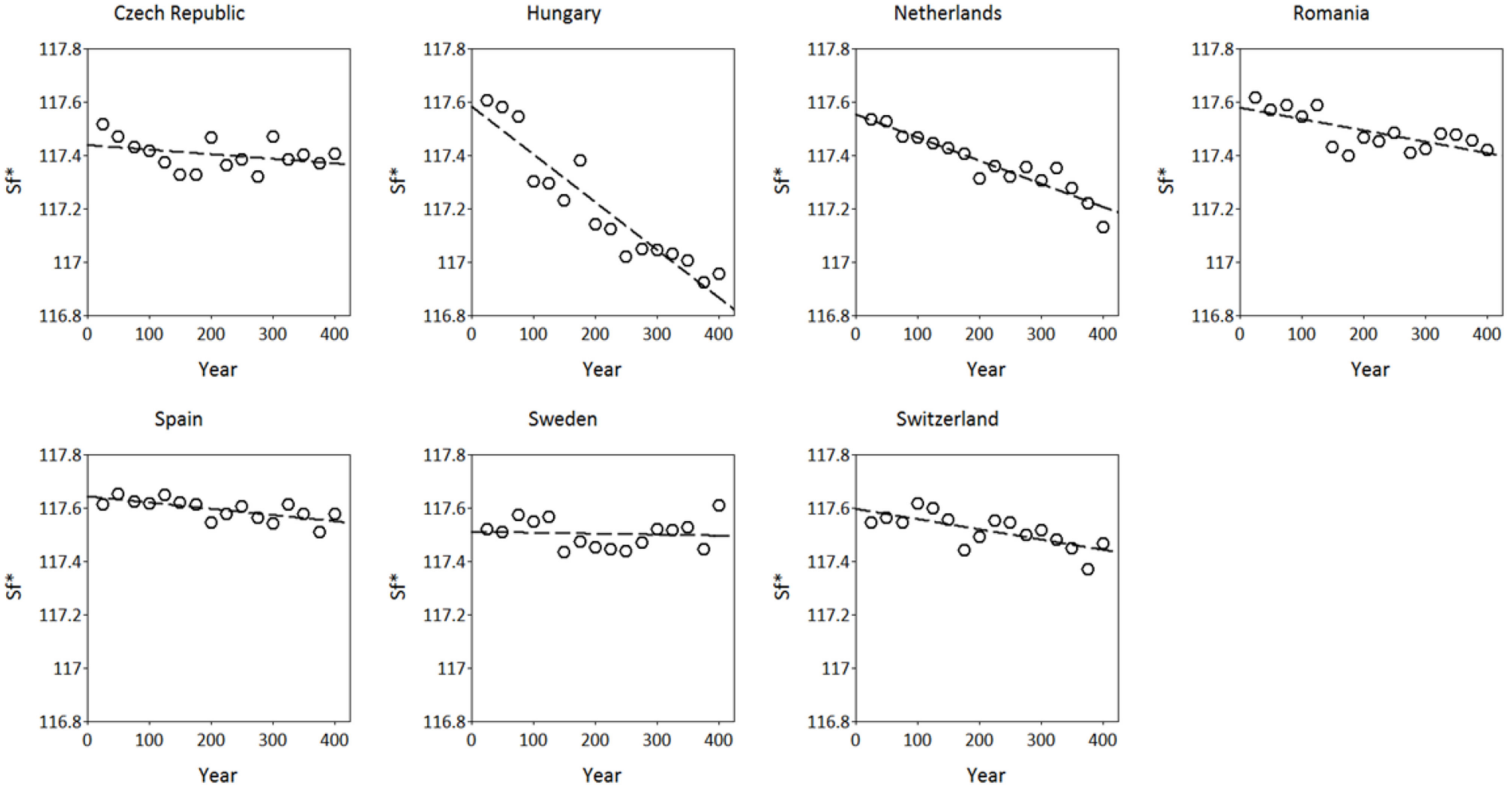
Adaptive response of critical state of forcing (S^*^_f_, in forcing units see Kramer, 1994b) over time after translocation of the Dutch population to the country indicated. The results presented are the mean of 5 replicate runs to account for genetic and environmental stochasticity.

The simulated adaptive response show clear trends with time for both S^*^_c_ and S^*^_f_ (Figures 8, 9). Interestingly, the model shows for the Netherlands a reduction of both S^*^_c_ and S^*^_f_ over time despite the fact that the site of origin and site of translocation were the same. This could indicate that the numerical method to estimate the model parameters can be improved by the genetic system applied in the ForGEM model. It could also be due to the fact that the model parameters were estimated based on observed temperature series, whereas the simulations presented here are based on output of the Hadley Global Circulation Model without additional CO_2_ forcing (Pope et al., 2000).

**Figure 10:**
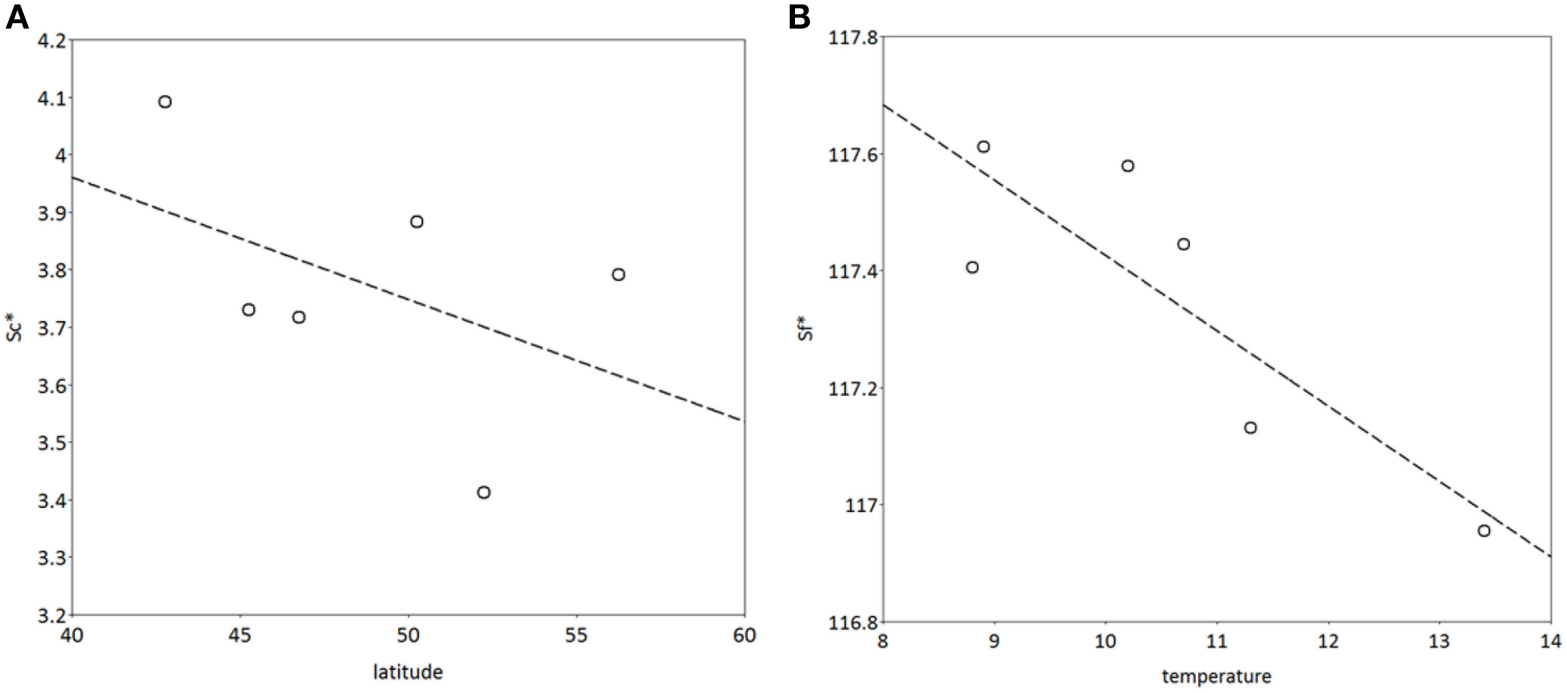
**(A)** Latitudinal cline of critical state of chilling (S^*^_c_), **(B)** dependency of critical state of forcing (S^*^_f_) on average temperature at the sites (see Table 2) after 400 years of simulation. The model was initialized with the same values for both S^*^_c_ and S^*^_f_ at all locations. The results presented are the mean of 5 replicate runs to account for genetic and environmental stochasticity. Note that the sites in Hungary and Switzerland have the same latitude, and the sites in Romania and Switzerland have the same average temperature (Table 2).

**Figure 11:**
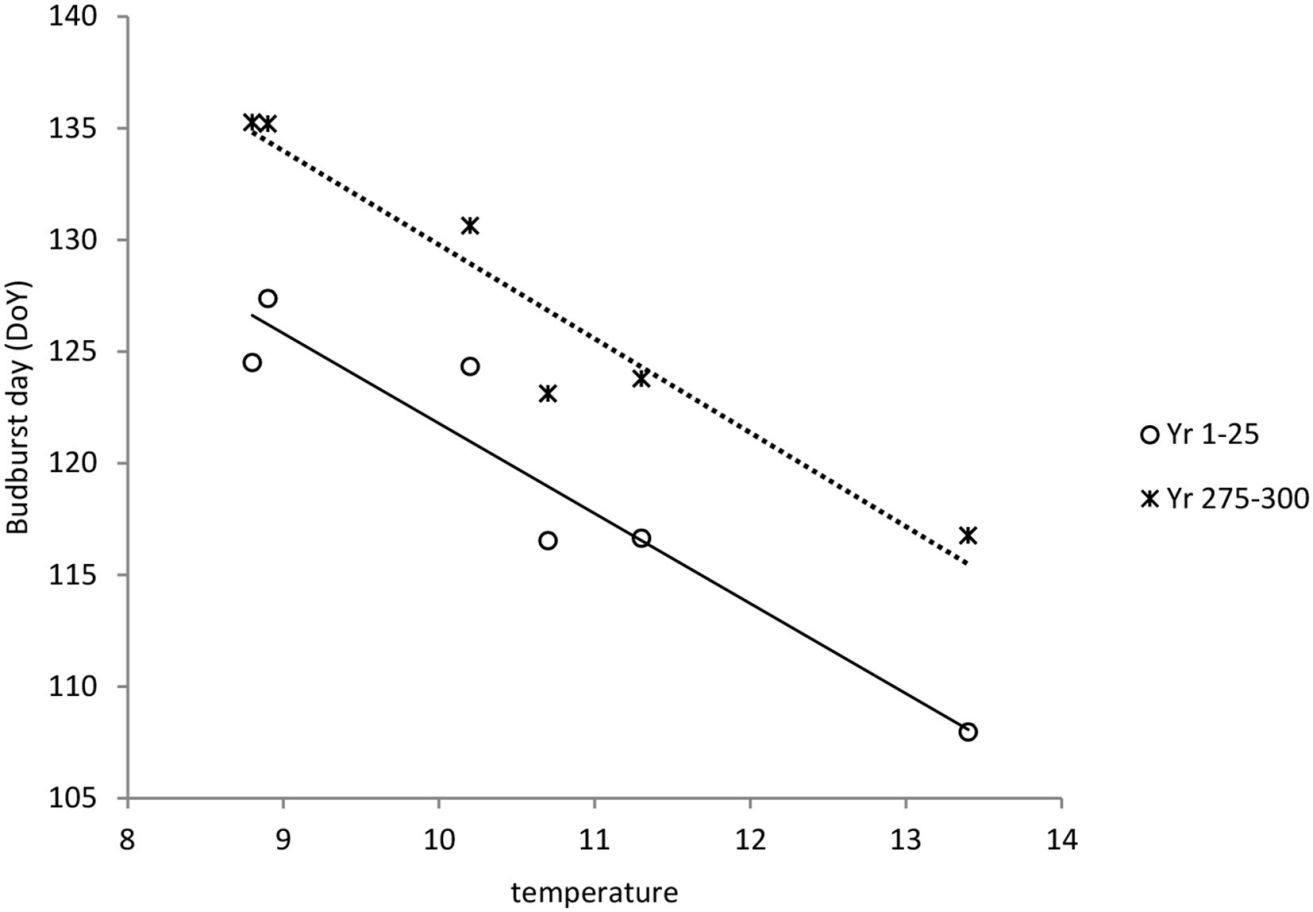
**Dependency of bud burst day on average temperature at the sites (see Table 2) both at the start and at the end of the simulation**. Average bud burst days are presented over 25 years to account for variability in the daily temperatures which are input in the ForGEM model. The results presented are the mean of 5 replicate runs to account for genetic and environmental stochasticity.

S^*^_c_ shows a rather clear trend with latitude after 300 years of simulation (Figure 10A), though with much scatter, whilst for S^*^_f_ the trend with latitude is very weak (results not shown). Vice versa, shows S^*^_f_ a clear trend with the average temperature at the simulated sites (Figure 10B), whilst that response is weak for S^*^_c_ (results not shown). The response of the bud burst day to average site temperature is a delay in bud burst day at the end of the simulation compared to the response at the start of the simulation (Figure 11). The penalty on a loss of foliage and flowers due to late night frost is probably too high.

## Discussion and conclusions

Dynamic global vegetation models assume a unique set of parameter values to characterize a plant functional type. At the global scale, the interest is in predicting shifts of the boundaries between plant functional types. It is unlikely that genetic processes determine the rate of change of boundaries between major vegetation zones under the influence of climate change, however, it is affected by adaptive capacity of the species. Also in the center of the species area, adaptive capacity may have an important effect on the rate of adaptation of resource acquisition and therefore competitive ability of the species, and on the response to extreme events.

To accommodate for local adaptations, an approach is presented to incorporate adaptive responses by evolutionary processes in an individual tree model. This approach was implemented in the ForGEM model and applied at a range of seven beech sites throughout Europe. For these sites, the distribution of the critical values for chilling and forcing, S^*^_c_ and S^*^_f_ respectively, were allowed to change in the population based on changes in frequencies of the alleles that determine the values of these parameters. The changes in allele frequency are the consequence of differential mortality because of different phenological parameter values of individual trees. Clear adaptive responses of both S^*^_c_ and S^*^_f_ were found in space and time despite the fact that the differences in average temperature between the sites of translocation and origin (Netherlands) was small (ranging from 2.5°C colder to 2.1°C warmer, see Table 2). In earlier studies with applications of the ForGEM model with a much larger temperature differences (+4°C to +6°C compared to a reference) we found that, firstly, genetic adaptation of forest trees is possible for important adaptive traits as phenology and water use within two to three stand rotations (interval between harvests); secondly, the rate of response of adaptive traits to climate change is strongly affected by forest management (Kramer and van der Werf, 2010).

The currently on-going whole genome studies will vastly increase the rate at which associations between quantitative trait loci and candidate genes and functional traits are found. Therefore, a large amount of directly useable genetic information is likely to emerge in the near future for many economically important tree species (Neale and Kremer, 2011). That will improve the initialization of the genetic system in ForGEM for local populations and particular traits, and thereby increase the accuracy of the adaptive responses to climate change for those populations and traits. We conclude that it is now feasible and necessary to include genetic processes in climate change assessment studies, based on these new and upcoming genetic insights in combination with the observed findings that different provenances of the same species of trees can strongly differ in their response to a similar change in the climate (Mátyás, 1996). Individual-based models incorporating genetic processes are essential for such analyses, as both climate envelop models and process-based models may-over estimate local extinction because these models do not include the genetic processes that allow trees to adapt to local conditions (Kramer and van der Werf, 2010).

### Conflict of interest statement

The authors declare that the research was conducted in the absence of any commercial or financial relationships that could be construed as a potential conflict of interest.

## Footnotes

^1^Inter-Sectoral Impact Model Intercomparison Project. Available online at: https://www.pik-potsdam.de/research/climate-impacts-and-vulnerabilities/research/rd2-cross-cutting-activities/isi-mip/data-archive.
