# Supplementary material for: Bring in the genes: genetic-ecophysiological modeling of the adaptive response of trees to environmental change. With application to the annual cycle
Source: Front Plant Sci. 2015 Jan 13;5:742. doi: 10.3389/fpls.2014.00742 (PMC4292233; doi:10.3389/fpls.2014.00742)
Supplement: Supplementary file 1 [file DataSheet1.DOCX]

1. Appendices.

Appendix A. Initializing allelic effects, allelic frequencies and phenotypic values

The approach followed in ForGEM to obtain the observed mean phenotypic value is:

- determine the U-shaped distribution of initial allele frequencies
- calculate mean and variance under the constraint of the U-shaped distribution of allele frequencies
- assign initially arbitrary allelic effects of *i* = +1 and *j* = -1 to each of the alleles *i* and *j* of all di-allelic loci
- scale allelic effects such that the distribution of phenotypic values over all possible genotypes is normalised (mean equals zero, variance equals unity)
- add the mean and multiply with the additive genetic variance of the functional trait in question

The equilibrium frequency distribution of alleles of neutrals traits is used to obtain the initial allele frequency distribution for the genetic model parameters. This distribution can be described by (Nei, 1987, p. 367):

$$\varphi\left( x \right)= \frac{\Gamma\left( M+ M^{'} \right)}{\Gamma\left( M)+ {\Gamma(M}^{'} \right)}\cdot\left( \left( 1-x \right)^{M-1}\cdot x^{M^{'}-1} \right)$$

With:

*M = 4·N_e_ ·v* 
*M' = M / (k -1)*

*N_e_*  effective population size

*v*  mutation rate per locus and per generation

*k* the number of alleles per locus

*Γ()* gamma function

*M* can also be estimated from the average heterozygosity (*H*). If a large number of loci are examined, then: *M = H/(1-H).* To arrive at initial allele frequencies for an actual number of loci (e.g. 5), the cumulative distribution of φ(x) is calculated and the allele frequencies for the actual number of loci are determined at the quantile values of the cumulative distribution. i.e. every 20% quantile in case of 5 loci. To obtain the cumulative distribution of *φ(x)*, *φ(x)dx* is numerically integrated between 0 and 1 (extreme are excluded because *φ(x)*→∞ when x→0 or x→1 ):

$$\int_{0+}^{1-} \varphi\left( x \right)dx\approx\sum_{x=0.0000001}^{0.999999} \varphi\left( x \right)\Delta x \approx1$$

To compute the allele frequency for a given number of loci, the inverse of the integral of φ(x) is required, where φ(x) is the distribution of allele frequencies of all loci in a population. This inverse can be obtained by linear interpolation after evaluating φ(x) over a large number of x-values. In case *H* = 0.25 and *k* = 2, the frequencies are 0.006, 0.044, 0.141, 0.299, 0.499 for a 5 locus, 2 allele genetic system. In this way, 5 allelic frequencies are obtained that take into account the natural distribution of frequencies, with many of loci with low (or high) allele frequencies and few loci with allele frequencies around 0.5.

Based on this initial allele frequency distribution, the mean and variance of a genotype become:

$$m=\sum_{l=1}^{nLoci} pi+qj$$

$$var=\sum_{l=1}^{nLoci} {p\left( i-m \right)}^{2}+q\left( j-m \right)^{2}$$

Initials arbitrary allelic effects of *i* = +1 and *j* = -1 are assigned to each of the alleles *i* and *j* of all di-allelic loci. The following steps are made to arrive at allelic effects with mean of zero and a variance of unity for the whole population. First, we make expectations zero by offset, *c*, such that *m – c = 0* and sum of individual allelic effects.

$$m= \sum_{l=1}^{nLoci} p\left( i-c \right)+q\left( j+c \right)=0$$

$$=>\sum_{l=1}^{nLoci} pi- \sum_{l=1}^{nLoci} pc+\sum_{l=1}^{nLoci} qj+\sum_{l=1}^{nLoci} qc=0$$

$$=>\sum_{l=1}^{nLoci} \left( pi+qj \right)-c\sum_{l=1}^{nLoci} \left( p-q \right)=0$$

$$=>m-c\sum_{l=1}^{nLoci} \left( p-q \right)=0$$

$$=>c=\frac{m}{\sum_{l=1}^{nLoci} \left( p-q \right)}$$

$$var=\sum_{l=1}^{nLoci} {p\left( i-m-c \right)}^{2}+q\left( j-m+c \right)^{2}$$

$$=> var=\sum_{l=1}^{nLoci} p({i-c)}^{2}+q({j+c)}^{2}$$

This leads to a large number of possible allelic values. Arbitrarily, the first combination of allelic effects that yield the lowest expectancy (*m*) is selected. This is $\left( i-c \right)$, thus the standardized allelic effect, *e*, with $m_{e}=0$and ${var}_{e}=1$ is described by:

$$e=\frac{i-c}{\sqrt{var}}$$

See Table A1 for a numeric example for a 5 locus, di-allele genetic system.

Table A1. Example of vectors with allele frequency and effects for a 5-locus di-allele genetic system. ***p***: allele frequency; ***i*** *or* ***j:*** initial arbitrary effects of opposite sign but equal in value;  ***i-c*** or ***j+c***: centralized effects such that mean equals zero; allelic effects with lowest expectancy; standardized allelic effects (***e***) such that variance equals zero. *m*: mean of allele effects, *var*: variance of allele effects, *c* $\left( = \frac{m}{\sum_{l=1}^{nLoci} \left( p-q \right)} \right)$: scalar to attain *m=0.*

| Locus | Allele | p | initial effects:  *(i, j)* | centralized effects:  *(i-c, j+c)* | standardized effects:  *(i-c, j+c)/√(var)* |
| --- | --- | --- | --- | --- | --- |
| 1 | A | 0.005 | 1 | 1.080 | 0.489 |
| 2 | B | 0.043 | -1 | -0.920 | -0.417 |
| 3 | C | 0.138 | -1 | -0.920 | -0.417 |
| 4 | D | 0.298 | 1 | 1.080 | 0.489 |
| 5 | E | 0.500 | -1 | -0.920 | -0.417 |
| 1 | a | 0.995 | -1 | -1.080 | -0.489 |
| 2 | b | 0.957 | 1 | 0.920 | 0.417 |
| 3 | c | 0.862 | 1 | 0.920 | 0.417 |
| 4 | d | 0.702 | -1 | -1.080 | -0.489 |
| 5 | e | 0.500 | 1 | 0.920 | 0.417 |
|  |  | *m* | 0.243 | 0.000 | 0.000 |
|  |  | *var* | 5.000 | 4.872 | 1.000 |
|  |  | *c* | -0.08031331 |  |  |

With the standardized allele effects ***e*** (last column in table A1) and the probabilities ***p*** (*p_a_, p_b_, ...p_E_*) the phenotypic value for that trait for individual trees to initialize the stand are calculated in the ForGEM model as follows. With: *h^2^* : heritability; *V_g_* **:** genetic variance; *V_e_* **:** environmental variance; *V_t_* **:** total variance (=*V_g_* + *V_e_*).

1. Calculate the genetic variance *V_g_* and environmental variance *V_e_* from the total variance *V_t_* and heritability *h^2^: V_g_ = h^2^ x V_t_* and *V_e_ = V_t_ - V_g_*. *h*^2^ and *V_t_* should thus be known for the trait.
2. Multiply standardized allelic effects ***e*** with sqrt(*V_g_*) to obtain allele effects ***E*** having expected variance of *V_g_*.
3. Determine genotypes of individual trees by randomly sampling 2 gametes for each locus using the probabilities ***p***.
4. The phenotypic value for the trait is obtained by summing the allelic effects ***E*** over all loci and alleles representing the trait, adding the overall population mean, and adding the environmental deviate with variance *V_e_* and expectation of 0.
5. If there are also observations of the trait on individual trees, the phenotypic values and alleles obtained in 4. are reassigned to closest matching tree. *E.g.*, if phenological observations on individual trees are available, the tree with earliest bud burst obtains the set of alleles resulting in the earliest bud burst, i.e. the genotype with the most alleles with negative effects. If such observations are not available, phenotypic values are assigned randomly over the trees of the stand.

Appendix B. Gene flow by pollen dispersal

The probability that gametes of a mother tree *M_i_* and father tree *F_j_* meet can be estimated by the fraction pollen of *F_j_* that arrive at the position of *M_i_*, relative to the contribution to all other known and unknown father trees. The amount of pollen of any father tree arriving at the position of a mother tree depends on the amount of pollen produced by the father tree, the distance between the target mother tree and all possible father trees, the wind direction relative to the orientation between the mother and the father, and the overlap in flowering phenology between the father and the mother trees. The general equation for the decline of the amount of pollen with distance is ([Degen 1996](#_ENREF_5)):

$$y= y_{0}e^{-bd}$$

With:

| *b* | slope parameter indicating the rate of decline of the amount of pollen |
| --- | --- |
| *d* | distance from father tree |
| *y_0_* | maximum number of pollen produced by the father tree, at *d = 0* |

The processes affecting these 3 parameters are described in this section. It is thereby assumed that there is no incompatibility between genotypes.

The direction of the wind affects the slope parameter, *b*, whereas the overlap in flowering phenology between the target mother tree *M_i_* and *k* potential father trees determines which portion of the pollen emitted by the father tree *F_j_* can actually pollinate a given flowering mother tree. Thus, the fraction of *F_j_* pollen arriving at position *M_i_* can be described as:

$$P\left( M_{i}, F_{j} \right)=\frac{y_{0}\left( F_{i} \right) \cdot e^{-b\left( M_{i}, F_{j} \right) \cdot d\left( M_{i}, F_{j} \right)} \cdot t\left( M_{i}, F_{j} \right)}{\sum_{k} \left( y_{0}\left( F_{i} \right) \cdot e^{-b\left( M_{i}, F_{j} \right) \cdot d\left( M_{i}, F_{j} \right)} \cdot t\left( M_{i}, F_{j} \right) \right)+E_{M_{i}}}$$

With:

| *b(M_i_,F_j_)* | | slope parameter effected by of wind direction |
| --- | --- | --- |
| *d(M_i_,F_j_)* | distance between mother tree *M_i_* and father tree *F_j_* | |
| *y_0_(F_j_)* | amount of pollen of father tree *F_j_* at distance *d= 0* | |
| *t(M_i_,F_j_)* | effect of phenology, *i.e.* overlap in flowering phenology | |
| *E_Mi_* | amount of external pollen arriving at mother tree *M_i_* | |
|  |  | |

The effect of wind direction on the slope parameter, *b(M_i_,F_j_),* is calculated as follows:

$$b\left( M_{i}, F_{j} \right)= b_{0}+m\cdot cos\left( \alpha_{w}-\alpha\right)$$

With:

| *b(M_i_,F_j_)* | direction-dependent slope parameter |
| --- | --- |
| *b_0_* | slope parameter when *cos(x) = 0*, *i.e.* at wind direction perpendicular to direction of *M_i_* to *F_j_* tree |
| *α_w_* | main wind direction |
| *α* | direction from *F_j_* tree to *M_i_* tree |
| *m* | magnitude parameter |

The distance between parental trees, *d(M_i_, F_j_)*, is based on the Euclidean distance between mother tree, *i,* and father tree, *j* :

$$d\left( M_{i}, F_{j} \right)=\sqrt{\left( x_{i} - x_{j} \right)^{2}+\left( y_{i} -y_{j} \right)^{2}}$$

With *x* and *y* indicating the *x*- and *y-* co-ordinates of the parent trees.

The effect of phenology on flowering overlap between mother and father trees, *t(M_i_,F_j_)* is calculated as follows:

$$t\left( M_{i}, F_{j} \right)=\left\{ \begin{aligned} \left| {tM}_{i}-{tF}_{j} \right|\leq t_{FL}\to\frac{t_{FL}-\left| {tM}_{i}-{tF}_{j} \right|}{t_{FL}} \\ \left| {tM}_{i}-{tF}_{j} \right|>t_{FL}\to0 \end{aligned} \right.$$

With:

| *t(M_i_, F_j_)* | fraction of overlap of flowering between *M_i_* and *F_j_* trees, relative to the flowering duration of the *M_i_* tree |
| --- | --- |
| *tFL* | duration of flowering of trees |
| *tM_i_* | timing of flowering of mother tree *M_i_* |
| *tF_j_* | timing of flowering of father tree *F_j_* |

It is assumed that the duration of flowering of trees, *tFL*, is the same for all genotypes. The user can also define the fraction of pollen from sources outside the population that is being simulated. It is assumed that the genetic composition of these external pollen is the same as that of the population with which the model was initialised.

Appendix C. Mortality

Mortality is implemented as the probability that a tree will die during the time step under consideration. For any time step, the combined mortality chance is compared to a random sample from a uniform distribution. If the sample is smaller than the probability of mortality, the tree is considered to die and removed from the tree list. Mortality is the combined probability of mortality due to the following causes: reserves are completely depleted; the tree is outcompeted by others; self-thinning; age; storm; frost; (optionally random mortality to reduce number of saplings).

The overall probability of mortality, *P*, is calculated according to:

$$P=1- \prod_{i} \left( {1-P}_{i} \right)$$

With *P_i_* the probability mortality due to cause *i*. The different causes of mortality, *P_i_* , are determined as follows:

- if the reserves of a tree are depleted, it receives a 100% mortality probability.
- if the ambient temperature exceeds the level of frost hardiness to which the tree is acclimated. This frost event kills seedlings and saplings less than 2m in height. Adult trees lose all their foliage and flowers at such temperatures during the frost sensitive period following bud burst. Such frost event not immediately kills adults but may cause that reserves are depleted required to build-up new foliage which then causes the death of the tree.
- if due to competition crowns of adjacent tree overlap to such a degree that the edge of a crown reaches the stem of the suppressed tree, then this tree receives a 100% mortality probability. See below in section ‘Crown volume and –radius’ for the description of increment of crown radius and overlap of crowns between adjacent trees.
- if the maximum number of trees that can be supported on the plot area is exceeded, calculated over all individual trees of all tree species, exceeds the maximum number of trees as determined by the -2/3 self-thinning rule from Reinecke (in ([Zeide 1987](#_ENREF_30))). This self-thinning rule is applied in the ForGEM model to seedlings and saplings less than 2m in height only.

$$n_{max}={10}^{\left( \theta\cdot{log}_{10}\left( \bar{W} \right) \right)}$$

- if the tree approaches its maximum age. Age dependent mortality is based upon a Weibull distribution function. It depends on the species-specific maximum age, at which 95% of the trees are dead, and a period before that maximum age at which the population is 95% alive, but begins to decline. The cumulative density function of a Weibull distribution takes the following form:

${CDF}_{Weibull}={1- exp}^{{-\left( \frac{x}{\alpha} \right)}^{\gamma}}$

$\alpha=\frac{x_{start}}{-{ln\left( 1-v_{start} \right)}^{\frac{1}{\gamma}}}$

$\gamma=\frac{ln\left( \frac{ln\left( 1-v_{max} \right)}{ln\left( 1-v_{start} \right)} \right)}{ln\left( \frac{1-x_{max}}{1-x_{start}} \right)}$

Thus, the probabilities of death are such that 5% of the population is dead at age *x_start_* and 95% is dead at the maximum tree age.
